# DNA Methylation in Solid Tumors: Functions and Methods of Detection

**DOI:** 10.3390/ijms22084247

**Published:** 2021-04-19

**Authors:** Andrea Martisova, Jitka Holcakova, Nasim Izadi, Ravery Sebuyoya, Roman Hrstka, Martin Bartosik

**Affiliations:** Masaryk Memorial Cancer Institute, Zluty Kopec 7, 656 53 Brno, Czech Republic; andrea.martisova@mou.cz (A.M.); holcakova@mou.cz (J.H.); izadi.nasim@gmail.com (N.I.); ravery.sebuyoya@mou.cz (R.S.); hrstka@mou.cz (R.H.)

**Keywords:** DNA methylation, epigenetic modification, tumor, tumorigenesis, bisulfite conversion, restriction enzyme, DNA biosensor

## Abstract

DNA methylation, i.e., addition of methyl group to 5′-carbon of cytosine residues in CpG dinucleotides, is an important epigenetic modification regulating gene expression, and thus implied in many cellular processes. Deregulation of DNA methylation is strongly associated with onset of various diseases, including cancer. Here, we review how DNA methylation affects carcinogenesis process and give examples of solid tumors where aberrant DNA methylation is often present. We explain principles of methods developed for DNA methylation analysis at both single gene and whole genome level, based on (i) sodium bisulfite conversion, (ii) methylation-sensitive restriction enzymes, and (iii) interactions of 5-methylcytosine (5mC) with methyl-binding proteins or antibodies against 5mC. In addition to standard methods, we describe recent advances in next generation sequencing technologies applied to DNA methylation analysis, as well as in development of biosensors that represent their cheaper and faster alternatives. Most importantly, we highlight not only advantages, but also disadvantages and challenges of each method.

## 1. Introduction

DNA methylation in eukaryotes is an important epigenetic modification that regulates gene expression. It is vital in embryogenesis, affecting such processes as imprinting, X chromosome inactivation and silencing of repetitive DNA [1]. Its deregulation is associated with a range of human diseases, e.g., autoimmune diseases, metabolic disorders, neurological diseases, and cancer [2]. From a chemical point of view, DNA methylation involves an addition of a methyl group to the 5′-carbon of cytosine residues in CpG dinucleotides. CpG dinucleotides are distributed unevenly in mammalian genomes, clustered mainly in so-called CpG islands (CGIs) that are often found within gene promoters [3]. The main reason for this is that most 5-methylcytosines (5mCs) that are not clustered in CGIs are in default methylated and undergo over time deamination to thymine [4]. T’s are not recognized by the DNA repair machinery, which results in C to T transition as one of the most frequent mutation in humans. On the contrary, CGI promoters remain mostly unmethylated in the germline. Hence, when spontaneous deamination of C occurs at this site, it leads to the formation of uracil which is effectively removed by uracil-DNA glycosylase and CpG content is retained. CGI methylation in promoters of somatic cells leads to gene silencing either by direct inhibition of transcriptional factors, or indirectly via interaction of 5mCs with methyl-CpG-binding domain (MBD) proteins that repress transcription by recruiting enzymes that deacetylate histones [5].

The process of DNA methylation is mediated by different types of DNA methyltransferase enzymes (DNMTs). First, de novo DNA methyltransferases DNMT3a and DNMT3b create a methylation pattern on unmethylated DNA, which is then maintained during subsequent cell division by DNMT1 using hemimethylated strand. These enzymes are essential in embryogenesis and their loss of function is lethal [6]. A reverse process of demethylation may either occur passively by a loss of maintenance during cell division, or, as shown recently, actively via catalytic action of 10–11 translocation (TET) family of proteins that convert 5mC into 5-hydroxymethylcytosine (5hmC) [7].

Further below, we describe an impact of DNA methylation on carcinogenesis process, then illustrate principles of numerous methods that were developed to analyze DNA methylation status of both single genes and whole genome and present the newest advances in development of biosensors that could represent novel, cheaper and faster alternatives to current methods. Importantly, we highlight not only advantages, but also disadvantages and challenges that each method faces.

## 2. Role of DNA Methylation in Tumorigenesis

Given the critical role of regulatory pathways in which DNA methylation plays a part (especially at transcriptional level), aberrant DNA methylation has been generally associated with a range of human diseases, including cancer [8]. Many cancerous cells are characterized by abnormal methylation patterns compared to their physiological counterparts. It is well known that hypermethylation (i.e., higher rate of methylation) of promoter regions could lead to transcriptional silencing of certain tumor suppressor genes and also contributes to control of many regulatory proteins and enzymes. In contrast, hypomethylation (i.e., lower rate of methylation) during very early stages of cancer development is associated with promotion of genomic instability and cell transformation [9].

### 2.1. DNA Hypermethylation

Methylation status of CGIs is highly controlled and protected from DNMTs by several mechanisms including active demethylation, active transcription, replication timing and local chromatin structure. Problems with disruption of any of the mentioned mechanisms would grant DNMTs access to CGIs, in turn resulting in inactivation of many cellular pathways. The following examples show that DNA hypermethylation of known tumor suppressors is indeed involved in solid tumor carcinogenesis.

Cell cycle regulation genes, e.g., *p16^INK4a^*, *p15^INK4a^*, or retinoblastoma (*Rb*) commonly undergo DNA methylation-mediated silencing in a variety of cancers [10]. For instance, overexpression of tumor suppressor p16^INK4a^ has been involved in cellular senescence, aging and cell cycle progression [11]. This protein blocks cell cycle progression by inhibiting cyclin-dependent kinases 4 and 6, which mediate phosphorylation of retinoblastoma protein (pRB) in G1 phase, consequently blocking the cell cycle progression. Increased silencing of *p16^INK4^* due to DNA methylation leads to an increased level of pRB phosphorylation, and hence in unlimited cell proliferation [11]. Hypermethylated *p16^INK4a^* gene promoters were found in various solid tumors, such as hepatocellular carcinoma [12], lung [13], pancreatic, breast, cervical or bladder carcinomas, as well as melanomas and gliomas [14]. Moreover, in non-small cell lung carcinomas (NSCLC), loss of expression of *p16^INK4a^* is associated with poor survival [15].

Various critical genes related to DNA repair processes are also hypermethylated in tumor tissues. For instance, hypermethylation pattern of *BRCA1*, which is involved in DNA repair of double-stranded breaks and is essential for preserving genome integrity, was found in ovarian and breast carcinomas [16]. Silencing of genes involved in cell adhesion may lead to tumor aggressiveness and tumor progression [9], as was shown for *CD97*, *CTNNA1*, *DLC1*, and *HAPLN2* genes in which hypermethylation was associated with poorer survival of patients with ovarian cancer [17]. Furthermore, genes linked with cancer cell survival having proapoptotic functions were inactivated by hypermethylation, as in the case of *XAF1* (XIAP-associated factor 1) gene that is frequently hypermethylated in human urogenital cancers and contributes to the malignant progression of tumors [18], or *CASP8* (caspase 8) gene that was reported to be hypermethylated after glioblastoma multiforme relapse [19].

Interestingly, there is also a strong association between DNA methylation and short noncoding microRNAs. It was shown that microRNA biogenesis depends on DNA methylation and that methylated microRNAs are significantly enriched within cancerous phenotypes compared to unmethylated microRNAs [20].

### 2.2. DNA Hypomethylation

Overall gene methylation can also decrease as a result of global or gene wide hypomethylation effect. This phenomenon is accompanied by increased DNA damage, chromatin decondensation, and chromosome instability [21]. Repetitive sequences like long and short interspersed nuclear elements, and classical satellites, are normally heavily methylated [21,22]. The cells with abnormal histology due to aging or cancer, however, often show a noticeable loss of DNA methylation (hypomethylation) of these regions [21]. For instance, decrease in DNA methylation of human pericentromeric repeat sequence Satellite 2 (*SAT2*) is associated with accumulation of a large number of chromosome rearrangements and numerical alternation of chromosomes 1 and 16 [23]. In addition to repetitive sequences, hypomethylation was also observed in single-copy genes, including coding regions and introns. Single-copy genes like *MYC* and *RAS* can be activated by a decrease of 5mC content in the coding region as a consequence of global hypomethylation [21].

Moreover, DNA hypomethylation can activate genes which were silenced by hypermethylated promoter. For instance, genes from MAGEA gene cluster (melanoma-associated antigen A) are expressed only in spermatogonial cells and not in other somatic tissues. However, they can be again re-expressed in various cancers due to demethylation of their promoters [24]. Accordingly, abnormal expression of MAGEA genes is associated with increased aggressiveness and malignancy of breast, lung, and colorectal tumors [25]. Finally, distinct precancerous stages of cervical cancer due to HPV16 infection are associated with aberrant methylation profiles of viral *E6* gene promoter. E6 protein influences important cellular pathways by inhibiting the action of p53 protein, thus preventing cell cycle arrest and apoptosis and promoting carcinogenesis [26]. Our group showed that gradual demethylation of HPV *E6* promoter correlated with a progression of precancerous lesions [27].

## 3. Standard Methods of Analysis

DNA methylation can be analyzed using three major approaches, namely, bisulfite conversion, methylation-sensitive restriction enzymes, and affinity enrichment-based techniques, further discussed in following chapters. The choice of a particular method solely depends on the target of analyses. It is also important to know whether the targeted CpG dinucleotides are found in a CGI or whether they are individually dispersed. Although a great portion of DNA methylation studies is concerned with promoters containing CGIs, almost 45% of human gene promoters have only a few CpG sites. They usually control tissue-specific genes and methylation of CpGs inside these promoters was also shown to influence gene expression [28]. Moreover, if the targets of analysis are known and routinely analyzed, there is a number of validated and registered kits for methylation detection [29,30]. Moreover, the quality and quantity of input DNA, sensitivity and specificity of the chosen method, cost per sample, and availability of equipment plays an important role in the decision process. In the following pages, we will give an insight into particular techniques, their strengths and weaknesses, and offer a comprehensive picture that could make the decision process easier. We describe techniques that analyze DNA methylation on a single gene level as well as those targeting whole genome methylation.

### 3.1. Single Gene Techniques

#### 3.1.1. Bisulfite Conversion and Methods Based on Converted DNA

Bisulfite conversion is a three-step reaction whereby cytosines are, at low pH and high temperature, converted first to sulfonated cytosines, then deaminated to sulfonated uracils, and finally converted to uracils in a process of alkaline desulfonation. Subsequent PCR reaction amplifies uracils as thymines. In the case of 5mC, the deamination step is nearly two orders slower than for C. Therefore, after bisulfite conversion, 5mC’s remain unchanged and are amplified in PCR reaction as cytosines. Since double-stranded DNA (dsDNA) protects C from deamination, DNA must be first denatured and void of proteins to ensure successful conversion [31]. The main limitation of this method is fragmentation due to harsh conditions of the reaction, incomplete conversion which generates false-positive results and reduction of complexity of DNA from 4 to 3 bases. After the conversion DNA strands are no longer complementary, DNA is single-stranded and quite unstable; therefore, repeated freeze–thaw cycles are not recommended. The amount of input DNA is not such a huge limiting factor anymore since many kits working with ultralow DNA amounts are available. For instance, EZ DNA Methylation Direct Kit from Zymo Research offers the option of 50 pg as the lowest input from blood, tissue and cells without isolation. Different kits offer different properties and quality of converted DNA. Several studies compared such kits in terms of DNA fragmentation, degradation and conversion efficiency [32,33,34], making a good starting point in the decision-making process. To avoid using a kit, Wang et al. published a study in which they improved the bisulfite conversion workflow and tested the effect of various commonly used additives [35].

After successful conversion, one of the detection techniques follows. There are several golden standard methods that are often used, and the choice is made based on the type of analyzed locus and desired output. All of them include a PCR amplification step. The primer design might be a bit tricky and there are several rules which should be followed. As mentioned above, converted DNA has a reduced complexity, therefore long stretches of polyA and polyT are quite common, which complicates the primer design. Since DNA is not complementary anymore, it is important to choose a strand for which primers will be designed. They should contain homogenous base composition and, at the 3′-end, several T’s (C’s before conversion) to ensure amplification of only converted DNA. Primer length is usually around 30 nucleotides and often nested or semi-nested PCR is needed. It is difficult to amplify longer sequences and thus PCR amplicon should not be longer than 600 bp, depending on applied conversion kit.

##### Direct Sanger Sequencing

When compared with pyrosequencing or bisulfite-cloning sequencing (both discussed in next sections), it is a rather cost- and time-effective method. Its great advantage is single cytosine methylation evaluation with possible relative quantification. The quantification method is the same as for single nucleotide polymorphism (SNP) quantification when the ratio of peak heights from sequence chromatograms is used. This method is considered accurate for SNP quantification [36]. For DNA methylation analysis this method was deemed almost impossible due to high background noise, overscaled cytosine signals and base-caller artefacts. Nevertheless, Jiang and colleagues show that carefully optimized PCR combined with PCR product purification leads to quite clean chromatograms comparable with cloning and pyrosequencing [37]. The methylation rate is then calculated as C/(C+T). Furthermore, Brisotto et al. came with an improved strategy using 5′-end tailed primers containing cytosines and other bases in equal representation. This compensates for the lack of C inside the analyzed sequence and offers a way for normalization of C signals [38]. Therefore if only relative and approximate quantification is needed among a large number of samples this method could prove to be useful. However, optimization of PCR might be harder than expected and, in the end, even impossible.

##### Bisulfite-Cloning Sequencing

This method includes the cloning of amplified PCR products into plasmids, bacterial transformation, isolation of plasmid DNA from single colonies, and, finally, Sanger sequencing of plasmid DNA. For a detailed description of protocol and workflow, please refer to [39,40]. The biggest strength of this approach is that it gives methylation information about a single allele per clone. Major weakness is the time consuming and labor-intensive workflow. The choice of cloning approach can make a huge difference in the whole process. Since a single plasmid equals a single allele, a number of plasmids for each sample need to be sequenced for the result to be statistically valid. Even though it is a labor-intensive protocol, this method was used in the study of *Adenomatous polyposis coli* (*APC*) and *Rb* promoter methylation [41,42] and is still being used in recent cancer methylation studies [43,44]. Overall, bisulfite-cloning is a relatively expensive and time-consuming method, but it is the only method that offers well quantifiable single cytosine resolution except for high throughput sequencing and pyrosequencing.

##### Pyrosequencing

**Figure 1:**
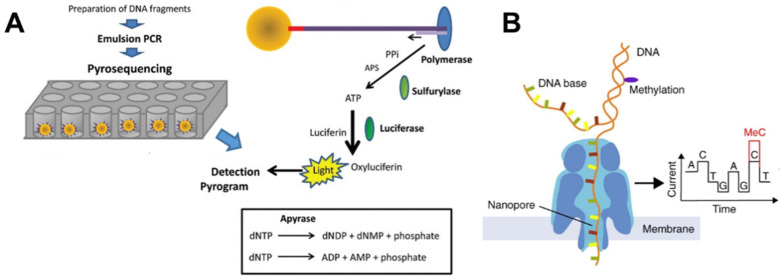
(**A**) Principle of pyrosequencing, comprising library preparation followed by emulsion PCR and sequencing itself. During sequencing, nucleotides are added one-by-one and are incorporated, according to the template sequence, by polymerase while releasing pyrophosphate (PPi). The PPi is then used by sulfurylase together with adenosine phosphosulfate (APS) to produce ATP. Luciferase then uses ATP in luciferin to oxyluciferin conversion. This reaction produces light that is detected in a pyrogram. Unused nucleotides are, after each incorporation cycle, degraded by apyrase. Adapted with permission from [74]; published by Taylor & Francis Ltd. (www.tandfonline.com, accessed on 19 April 2021). (**B**) Nanopore sequencing uses an electric field to move DNA toward a positive charge. DNA must pass through a narrow pore in the membrane (1.5 nm diameter). As the strand passes through the pore, individual bases block the opening, causing small changes in electrical conductivity of the membrane. Each base (including 5mC) has different properties based on its physical structure, making their identification possible. Reprinted with permission from [75]; published by Springer Nature.

Bisulfite pyrosequencing utilizes bisulfite conversion followed by PCR amplification with one biotinylated primer, immobilization of the amplicon on streptavidin beads, hybridization with sequencing primer, and subsequent sequencing (see Figure 1A for details) [45,46]. This method does not give information about allele-specific methylation as bisulfite-cloning sequencing, but rather gives an average methylation level of both alleles in a highly quantitative manner. The main advantage is that it generates background free chromatograms from which percentage of methylation is calculated as C/(C + T) with great precision. The biggest disadvantage of this technique is that it generates only short reads and that analyzed CpGs should be located close to the sequencing primer since increasing length of sequencing product (longer than 90–100 nt) leads to the decrease of the accuracy of sequencing data. Therefore, the quantification of more CpGs in longer stretches of DNA requires multiple reactions. Dupont et al. offered improved protocol with which they analyzed 10 CpGs in one run spanning up to 75 nt of DNA [47]. Furthermore, Kreutz et al. presented detailed protocol with an application on CpG analysis [48] and Delaney et al. published a protocol including example of data analysis [49]. Overall, this method offers great resolution per CpG in any type of methylation site with a straightforward assessment of methylation rate. However, it comes hand in hand with a high price for sequencing instrument and a higher price per measurement. A more detailed comparison with other methylation analysis techniques can be found in the review by Šestáková et al. [50]. From commercially available services, EpigenDx provides pyrosequencing analysis of gene-specific methylation on their pre-validated assays with over 7000 human, mouse and rat gene loci [51].

##### Methylation-Specific PCR (MS-PCR)

MS-PCR is a methylation analysis method suitable only for CGIs. Moreover, this analysis does not provide information about the methylation status of single cytosines. It requires two sets of primers—one for methylated and one for the unmethylated allele. With those primers, two sets of parallel PCR reactions are performed for each analyzed sample. PCR primers should anneal to identical sites, they should contain one to three CpGs and several T’s from converted C’s to ensure only converted DNA is amplified. Moreover, they should not differ in their melting temperature (Tm) by more than a few °C. A more detailed description of the workflow can be found in [52]. In the original version, PCR products were analyzed by gel electrophoresis, however nowadays Real-Time PCR is often used which allows for quantification using Ct values. The method utilizing TaqMan probes is called MethyLight [53] and it offers highly sensitive analysis while the SYBR Green approach is called MethylQuant and trades lower price for slightly less specific analysis than MethyLight [54,55]. The downside of this approach is that it cannot be utilized for sites with a low number of CpG and that primer design is quite challenging [50].

##### Methylation-Specific High-Resolution Melting (MS-HRM)

MS-HRM analysis is based on different Tm between C-G (3 H bonds) and A-T (2 H bonds) pairs. Melting analysis is performed after methylation-nonspecific PCR amplification. The temperature is gradually increased and PCR products from unmethylated allele containing A-T are dissociated at a lower temperature indicated by an abrupt drop in fluorescence when an intercalating dye (SYBR Green, Eva Green, SYTO9) is released from dsDNA. The PCR amplicon should be kept around 100 bp otherwise it may contain secondary structures which could interfere with the analysis. The whole protocol, including discussion of primer design, is well explained in Wojdacz et al. [56]. This method is relatively time effective when compared with other bisulfite conversion-based methods and distinguishes well between fully or partially methylated sites against unmethylated, meaning it is of great use when only a small portion of analyzed sample loci is methylated. The disadvantage is that it is impossible to analyze only a single CpG. This approach is semiquantitative if an unknown sample is compared with standards of known methylated vs unmethylated ratio [57]. Overall, MS-HRM offers a fast and relatively cost-effective estimation of methylation rate [50] and has a great use in the analysis of clinical samples [58].

##### Other Bisulfite Specific Methods

A popular technique is a combined bisulfite restriction analysis (COBRA), which is a technique that combines bisulfite conversion with restriction enzymes. After conversion and PCR amplification the product is digested with restriction enzymes (RE), resolved by gel electrophoresis and then it can be blotted to a membrane, hybridized with probes and quantified by phosphoimager (original approach from 1997) [59]. Bisulfite conversion either creates new restriction sites or retains the old ones. Bilichak and Kovalchuk offered a more detailed description with the list of possible REs [60]. This method has software support (Methyl-Typing) [61] and is still used nowadays—for example, it was used for the analysis of 3 key CpG sites in *ROR2* promoter in endometrial cancer [62]. The disadvantages of COBRA include a time-consuming protocol (conversion, RE digestion, and gel electrophoresis), limitation by available RE sites and generation of false-positive results by incomplete digestion.

Methylation-sensitive single-nucleotide primer extension (MS-SnuPE) was originally introduced in 1997, and it utilizes the idea from analysis of gene mutations [63]. Briefly, after conversion and PCR amplification the PCR product is used in second reaction with primers annealing inside the sequence ending with their 3′-end just before the analyzed CpG. Two parallel reactions are set with either ^32^P-dCTP or ^32^P-dTTP and Taq polymerase. The obtained products are resolved by electrophoresis and analyzed by phosphoimager [64,65,66]. The advantages of this method include single cytosine analysis without RE or sequencing combined with the fact that it is semi-quantitative and that more than one CpG can be analyzed per one reaction when utilizing multiplex strategy. The disadvantage is that each site requires two parallel reactions and includes radioactively labelled compounds. Nonetheless, a nonradioactive variant utilizing ion-pair reversed-phase high performance liquid chromatography (HPLC) also exists [67], but it requires an HPLC instrument. More recent works using this technique can be found in refs. [68,69].

Epityper (Agena Bioscience) is an analytical method that uses base-specific cleavage and MALDI-TOF MS (matrix assisted laser desorption/ionization time-of-flight mass spectrometry). It requires bisulfite PCR with primers outside analyzed CpGs with one of the primers tagged with T7 promoter followed by in vitro transcription, base-specific cleavage by RNAse A and MALDI-TOF MS [70,71,72]. The great advantage is that it allows analysis of several CpGs in one reaction in a span of 200–600 bp. Furthermore, it is highly reproducible, relatively fast, quantitative, and high throughput so it is a good choice for a large number of samples. However, it requires the availability of an expensive instrument and if polymorphism is present in the sequence of interest it can make the analysis more difficult. This method has been used in the detection of *XXYLT1* methylation in lung cancer [73].

#### 3.1.2. Methods Based on Methylation-Sensitive Restriction Enzymes (MSRE)

MSREs are unable to cleave DNA if their restriction site is methylated. Therefore, methylated DNA stays intact while unmethylated is digested by the RE. They can be used in combination with isoschizomer (enzymes with identical restriction site) insensitive to methylation. The most frequently used pair of enzymes is HpaII (MSRE) and MspI (insensitive isoschizomer) which cleave CCGG restriction site. This approach is often used for CGI analysis. Historically it was the original way of DNA methylation analysis from 1979 [76]. The great advantage of this approach is that it does not require bisulfite conversion, therefore lower DNA input is needed with a considerably easier primer design. The disadvantages, on the other hand, include the fact that only sites with available MSRE can be analyzed, and furthermore if the digestion is incomplete false-positive results are generated. According to the Šestáková et al. the price per sample is quite high [50]. Several detection techniques follow after the restriction enzyme digestion. The older approach involved Southern blot analysis and required input around 10 μg of DNA [77].

##### MSRE-PCR

A more widely used approach utilizes PCR amplification with primers flanking the restriction site. If the DNA is methylated, digestion does not proceed, leading to successful PCR amplification. Our group successfully applied this approach in determination of *E6* gene promoter methylation in human papillomavirus 16 isolated from cervical smears [27]. The modern approach utilizes Real-Time PCR. A great description of this method can be found in [78] where they address enzyme and template amount optimization so that nonspecific restriction and incomplete restriction are both minimized. The disadvantage is that at least 2 CpG RE sites should be present in an amplicon for the assay to be reliable [50]. Overall, this method is suitable for both methylated and unmethylated sites but with a higher frequency of CpG. Moreover, Zymo Research offers OneStep qMethyl-PCR kit which contains reagents and controls for quantitative detection which is ideal for screening several loci if one does not want to optimize their controls.

##### Methylation-Sensitive Multiplex Ligation-Dependent Probe Amplification (MS-MPLA)

MS-MPLA is a technique originally based on the HhaI enzyme which cuts GCGC sites with probes also containing this sequence. It is described in greater detail in [79,80]. The advantages of this approach include single cytosine resolution in a multiplex fashion without the need for bisulfite conversion. Moreover, it is semi-quantitative. Downsides of this approach include optimization and design of probes combined with the need for special ligase and capillary gel electrophoresis.

#### 3.1.3. Affinity Enrichment-Based Approaches

These are enrichment approaches that use either affinity purification of 5mC with MBD proteins [81] or immunoprecipitation with antibodies against 5mC (MeDIP, methylated DNA immunoprecipitation) [82]. Antibodies are better for denatured ssDNA with a low density of CpGs while MBDs bind dsDNA and are better for the enrichment of CGIs. There are several kits available and the whole approach is well-reviewed in [83]. Methylated-CpG Island Recovery assay (MIRA) is based on MBD2b/MDB3L1 complex which binds methylated dsDNA and is further described in [84]. After elution of enriched DNA sequences, a bisulfite conversion takes place followed by one of the already described techniques or the enriched DNA can be used in one of the genome-wide approaches described in following sections.

### 3.2. Whole-Genome Techniques

Under whole-genome techniques, we distinguish methods that determine global DNA methylation (see Section 3.2.1), and methods for genome-wide DNA methylation profiling (Section 3.2.2). Global DNA methylation assays do not analyze methylation of specific sequences but are rather used to determine total genomic content of 5mC. Estimation of total genomic methylation is used as a marker for biochemical changes such as nutrition, age or chemical exposure. Global methylation is also more resistant to changes than site-specific DNA methylation, and therefore its determination is more often used to study dynamic epigenome changes during embryonic development or tumorigenesis [9,85]. On the other hand, genome-wide DNA methylation profiling is used to analyze DNA methylation status of all CpG sites at the whole genome level (i.e., total DNA methylome), using analogous approaches that are used for single-gene analysis (described in Section 3.1).

#### 3.2.1. Methods for Determination of Global DNA Methylation

There are a number of molecular methods for testing global DNA methylation. While most methods target organisms with known genomic sequences, there are also some techniques that can be used for less common laboratory species without the knowledge of their genome. However, each of the global methods focuses on testing different regions or sequences in the genome, so a careful selection of an appropriate method for a given experiment should be considered.

##### Enzyme-Linked Immunosorbent Assay (ELISA)

One of the most commonly used methods for global DNA methylation assessment is ELISA. It is a simple and cost-effective method suitable for determining the overall level of DNA methylation. Usually, a competitive ELISA is used for the quantitative measurement of 5mC (and other cytosine modifications). The 5mC cytosine content of the unknown samples is determined by comparison with a predetermined 5mC standard curve. Since it is an assay based on relative quantification, positive and negative controls are essential. Methylated DNA is detected by specific antibodies and then quantified by reading the absorbance on a microplate spectrophotometer. The percentage of methylated DNA is proportional to the measured optical density. ELISA is a very robust method for DNA isolated from different species using different isolation techniques. Various ELISA kits are commercially available, but they vary widely in terms of input DNA requirements, sensitivity, protocol time, and efficiency, e.g., Global DNA Methylation ELISA (Cell Biolabs, San Diego, CA, USA), MethylFlash Global DNA Methylation ELISA Easy Kit (EpiGentek Group, Farmingdale, NY, USA), or Methylated DNA Quantification Kit (Abcam, Cambridge, UK). Main limitations of ELISA include lower specificity and cross-reactivity of used antibodies and that it provides only a rough estimate of global DNA methylation.

##### Mass Spectrometry

Mass spectrometry represents a useful technique for the detection of global DNA methylation. The most precise quantification of DNA is based on the determination of the absolute abundance of the different bases of the DNA and their modifications by using multiple reaction monitoring in a mass spectrometer (MS–MRM) coupled to nano-ultra HPLC [86]. Global methylation analysis by mass spectrometry is highly accurate, sensitive, and reproducible; moreover, the analysis runs across the entire genome, regardless of site or sequence. In addition, this method easily distinguishes between 5mC and other epigenetic modifications of DNA. The disadvantage is the technical complexity and the associated higher costs [86,87].

##### Luminometric Methylation Assay (LUMA)

LUMA is a fast, quantitative and highly reproducible method based on a combination of DNA digestion with methylation-sensitive restriction enzymes and pyrosequencing. The LUMA assay is used to analyze DNA methylation throughout the genome for various physiological and pathological conditions, including various aspects of cancer [88]. The principle of the assay is DNA digestion with methylation-sensitive (HpaII) or insensitive (MspI) restriction enzymes, followed by a polymerase-extended bioluminescence assay to quantify the extent of restriction cleavage. EcoRI digestion is used as an internal control. LUMA does not require comparison with a reference genome and as such is applicable to all organisms. Because no primary modification of genomic DNA is required, such as bisulfite conversion, the analysis is quite rapid [89]. However, the results of LUMA are affected by the DNA isolation technique plus the enzymatic cleavage is limited to the 5′-CCGG-′3 restriction sites, which make up only about 8% of all CpGs in the genome [90]. In addition, MspI also cleaves at sites with 5hmC modification and does not distinguish it from 5mC [91].

#### 3.2.2. Methods for Genome-Wide DNA Methylation Profiling

Individual approaches can be divided according to whether they analyze DNA methylation in the whole genome or only in selected loci or regions such as promoters or predicted highly methylated sequences.

##### DNA Methylation Arrays

One of the technologies for analysis of selected loci are microarrays, generally based on hybridization that allow analysis of thousands of sequences simultaneously with minimal DNA consumption. Arrays for total epigenome analysis are commercially available. The most used platforms comprise Illumina, which offers MethylationEPIC BeadChip, or Infinium HumanMethylation450K that use predesigned probes that recognize methylated and unmethylated cytosine in bisulfite-converted DNA. MethylationEPIC BeadChip provides comprehensive quantification of more than 850,000 CpG sites across all known genes including CpG islands, enhancers, open chromatin, transcription factor binding sites or miRNA promoter regions. Other platforms, such as Agilent’s Human DNA Methylation Microarrays, are based on the affinity isolation of methylated DNA (MeDIP). Methylated regions of a genomic DNA sample are pulled down with a monoclonal antibody against 5mC, labeled with cyanine 5, and hybridized to microarray probes. In parallel, control genomic DNA labeled with cyanine 3 is also hybridized on the microarray. Relative levels of DNA methylation are then determined as cyanine 5/cyanine 3 ratios [92]. Main limitation of microarrays is that they cover only a certain portion of total CpG sites within the human epigenome, usually defined by a manufacturer.

##### Next Generation Sequencing (NGS)

NGS offers the possibility of rapidly obtaining complex data on DNA methylation of any species with a mapped genome. This technology circumvents the distortion created by the use of specific probes, allele-specific differences, and amplifications that occur in microarray technology. One type is so-called Whole Genome Bisulfite Sequencing (WGBS) used mostly for sequencing of bisulfite-converted DNA, where each cytosine is analyzed. WGBS is a quantitative platform that is highly reproducible and accurate. However, the high cost of overall sequencing runs and the complexity of computational analysis and evaluation are limiting factors for its widespread use. Although this is a high-quality analysis, its throughput is relatively low, so RRBS (Reduced Representation Bisulfite Sequencing) is used more often. This approach reduces costs and increases throughput of samples at the expense of lower coverage. RRBS involves enrichment of CpG-rich regions (CGIs, promoters) in a genome by the cleavage with MspI and selection of DNA fragments by size (70–320 bp), prior to bisulfite conversion and sequencing [93]. The main advantages of RRBS are lower cost and less data congestion, but unlike WGBS and array technologies, it is less reproducible.

Another option is sequencing of enriched DNA, i.e., sequencing is preceded by MeDIP or MBD-Cap (termed MeDIP-seq or MBD-cap-seq), where the methylated DNA fragments are captured by a specific antibody or proteins, purified, amplified, sequenced and mapped according to the reference genome [94,95]. This method does not allow accurate identification of methylated sites, nevertheless, it can be used to estimate the level of methylation in a particular region. Similarly, sequencing can be combined with MSREs (MSRE-seq), using several methylation-sensitive REs (BstUI, HpaII, NotI, and SmaI) that cleave genomic DNA at various unmethylated restriction sites. Sequencing of cleaved DNA fragments, which are enriched in unmethylated CpG at their ends, allows for the identification of cleavage sites. Both of these methods, as well as their other modifications, have low resolution and genome coverage. However, they are cost-effective and do not require bisulfite treatment of DNA.

DNA cleavage and amplification are critical steps in many DNA sequencing techniques. However, these steps are not required in long read sequencing, also called third generation sequencing. These methods allow reading of nucleotide sequences of 10^4^–10^6^ bases at once. One of them is Single Molecule Real-Time sequencing (SMRT) that allows detection of methylated bases without bisulfite conversion, including 5mC, 5hmC as well as 6-methyladenine [96,97]. Because different modifications affect polymerase kinetics differently, SMRT monitors all modifications simultaneously. At the same time, it can be used for long reads and mapping of methylation patterns in highly repetitive genomic regions [98]. SMRT sequencing is mainly used for mapping bacterial genomes, where 6mA and 4mC modifications are densely represented, thus giving strong and reliable kinetic signals. On the other hand, this method is less sensitive to 5mC detection [99].

Finally, Nanopore sequencing as the fourth-generation DNA sequencing technology developed by Oxford Nanopore Technologies Ltd., is the most powerful method for rapid generation of long-read sequences without the need for PCR amplification or chemical labeling of the sample [100]. Nanopore sequencing uses electrophoresis to transport an unknown sample through an orifice of 10^−9^ m in diameter (Figure 1B). It identifies DNA methylation patterns when ssDNA is ratcheted through a biological nanopore, and records ion current deviations due to specific base modifications passing through the pore [101]. The main disadvantage of Nanopore sequencing, as with other long-read sequencing approaches, is the relatively high error rate ranging from 5 to 20% [102,103]. However, algorithms that convert the raw ion current signal into nucleotide sequences, as well as bioinformatics tools specialized or optimized for evaluating Nanopore sequencing, are constantly evolving and improving [104,105].

## 4. Biosensors for DNA Methylation Analysis

Despite an arsenal of standard or conventional techniques available for DNA methylation analysis, a huge effort is made to develop novel biomedical tools that would address the most common limitations of standard techniques, highlighted in Section 3. In recent years, many scientists have turned their attention towards the development of alternative assays in a biosensor format, i.e., solid-phase assays or devices that employ specific biorecognition elements on their surfaces to capture target analyte and a signal transducer that converts this biorecognition event into some type of a signal. Main advantages of these so-called DNA biosensors are their high sensitivity and specificity, affordability as they mostly use low-cost analytical instruments, simplicity and rapid processing times. Since we talk about DNA biosensors, biorecognition elements for both categories are almost exclusively made of complementary DNA capture probes that bind to methylated or unmethylated target DNA. To distinguish methylated from unmethylated DNAs, these biosensors also utilize one of the three major approaches mentioned in Section 3, i.e., sodium bisulfite conversion, MSREs or affinity-based strategy using antibodies/MBD proteins. Below, we divide biosensors into two main categories based on signal transduction: optical and electrochemical (EC) biosensors and illustrate their advantages and challenges on several examples.

### 4.1. Optical Biosensors

Optical biosensors are analytical tools that detect light produced when a target analyte is captured by biorecognition layer. Most optical biosensors use colorimetric (naked eye) or fluorescence detection; less frequent options include chemiluminescence, surface-enhanced Raman spectroscopy or surface plasmon resonance. They attracted attention of many authors due to their high sensitivity, affordability, reproducibility, and specificity. These sensors have shown success not only on model systems, but also cancer cell lines or even clinical samples for the detection of cancer biomarkers [106,107,108].

Colorimetric optical biosensors enable visual detection by naked eyes due to their ability to show color changes. They trade their user-friendliness and simplicity for somewhat lower sensitivity. For instance, Zheng’s group have developed a rapid colorimetric biosensor based on unmodified gold nanoparticles (AuNPs) to analyze methylation of *p53* gene fragment [109]. Methylated and unmethylated probes were treated with sodium bisulfite and then hybridized to a third probe complementary to originally unmethylated probe. This duplex formation, positive only in the case of 5mC absence, has been visually detected using differences in electrostatic attraction of ssDNA and duplex dsDNA to salt-induced aggregates of AuNPs. Duplex DNA from unmethylated probe exhibited lower attraction to AuNPs because of the negative charge of the phosphate backbone that repelled the negatively charged citrate on the surface of the AuNPs. Lower binding of duplex DNA translated into easier aggregation of AuNPs, changing color from red to purple. Authors even applied their biosensor into real human samples, unfortunately only by spiking of the probes into human samples without specifying what type of the sample they used. Moreover, limit of detection (LOD) of this assay was rather high, in nanomolar range, rendering its application in clinical samples improbable.

**Figure 2:**
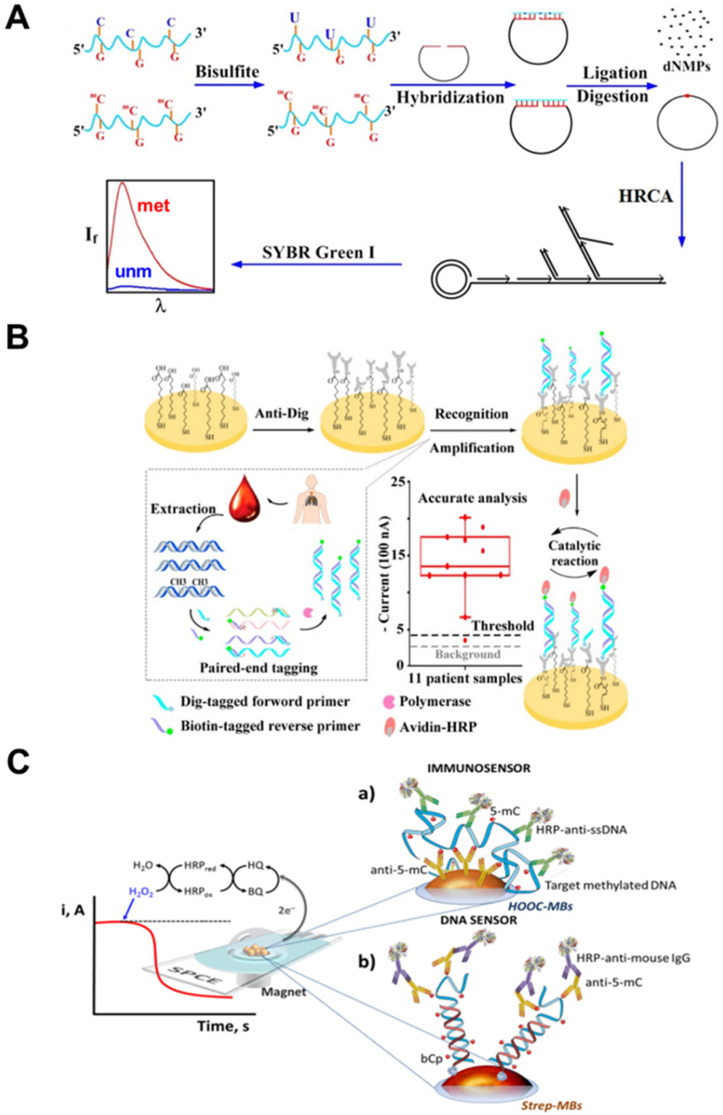
Examples of fluorescent and EC biosensors for analysis of DNA methylation. (**A**) Fluorescent biosensor utilizing bisulfite conversion and HRCA reaction applied to lung cancer cell lines. Reprinted with permission from [110]. Copyright (2012) American Chemical Society. (**B**) EC biosensor based on asymmetric MS-PCR using biotin-labeled reverse primer and HRP signal monitoring applied to plasma of NSCLC patients. Reprinted with permission from [119]; published by Royal Society of Chemistry. (**C**) EC immunosensor (**a**) and DNA sensor (**b**) utilizing anti-5mC antibodies and HRP monitoring applied to colorectal tissue samples and human serum. Reprinted with permission from [132]; published by Springer Nature.

Sensitivity can be greatly improved using optical biosensors based on fluorescence detection. In study by Zhang et al. target DNA was treated with sodium bisulfite that converted C to U while 5mC remained unchanged [110] (Figure 2A). Afterwards, methylation-specific linear padlock probe was applied to specifically circularize only in a presence of methylated DNA due to hybridization between G and 5mCs, but not unmethylated DNA having U instead of 5mC. The circularized product was amplified by ligation-mediated hyperbranched rolling circle amplification (HRCA), whose products could be easily detected using SYBR green I and a standard fluorimeter. This assay was applied to determine methylation status of six CGIs in the *p16* promoter region of human lung cancer cell lines H157 (non-small cell lung cancer cell lines) and H209 (small-cell lung cancer cell line), but not to clinical samples.

Extremely sensitive fluorescent biosensor was developed by Zhang et al. even without using bisulfite reaction and PCR. Authors applied HpaII to digest unmethylated DNA, while undigested methylated DNA hybridized to dual-labeled probe (with Cy5 and biotin) having abasic site in the middle. This duplex was then bound to streptavidin magnetic beads and cleaved at the abasic site by endo IV to release Cy5 into the solution. Liberated methylated DNA could again bind to unbound probes for subsequent rounds of endo IV cleavage, which further released Cy5 fluorophores. Due to the catalytic nature of the process, LOD of 7.3 × 10^−17^ M was reached; moreover, biosensor could recognize as low as 0.01% methylation level, and was applied to analyze DNA methylation of *p16* gene in various cancer cell lines, such as hepatoma cell line (Hep G2), breast cancer cell lines (MDA-MB-231 and MCF-7), cervical carcinoma cell line (HeLa), human lung adenocarcinoma cell line (A549), and normal human epithelial mammary cell line (MCF-10A). Although the protocol was rather complex and required methylated probe, increasing overall price of the assay, it is indeed very interesting strategy that would be at least worth to test on clinical samples [111]. More examples of optical biosensors can be found in Table 1.

**Table 1:** Examples of optical and electrochemical biosensors.

Biosensor Type	LOD	Target Gene	Sample Type	Reference
***Optical***				
Hyperbranched rolling circle amplification-based fluorescent biosensor	0.8 fM	*p16*	H157 (non-small cell lung cancer cell lines), H209 (small-cell lung cancer cell line)	[110]
Dual enzyme/dual-labeled fluorescent probes	7.3 × 10^−17^ M	*p16*	Hep G2, MDA-MB231, MCF-7, HeLa, A549, MCF-10A	[111]
QD-FRET ^1^	N/A	*PCDHGB6*, *HOXA9*, *RASSF1A*	Lung adenocarcinoma and non-tumor tissues	[112]
LCR-AuNP ^2^ colorimetric	0.01 fM	N/A	Blood of healthy volunteer	[113]
LCR-mediated QD- based FRET	1 aM	*PL6*	Lung cancer cell lines	[114]
RE-EXPAR ^3^	200 aM	*Septin 9*	HCT116 colorectal cancer cell lines	[115]
MELZA ^4^	N/A	Androgen receptor gene promoter	LNCaP, PC3, Du145 cells and whole blood cells	[116]
Methylation-sensitive cleavage-based PG-EXPA ^5^	8.6 × 10^−5^ U/mL	N/A	Human serum ^6^	[117]
Label free colorimetric and fluorimetric assay	9.4 × 10^−10^ M	N/A	Human plasma	[118]
***Electrochemical***				
Sequential discrimination-amplification strategy	3 pg	N/A	Plasma from lung cancer patients	[119]
Smart coupling immuno-magnetic beads assay	4 pM for 5mC; 100 pM for 5hmC	*RASSF1A*, *MGMT*	Cancer cells, paraffin-embedded colorectal tissues (MGMT), serum samples from breast and lung cancer patients	[120]
DNA-graphene affinity interaction assay	N/A	*FAM134B*	ESCC ^7^ cell lines and patients	[121]
Label-free electrochemical assay	N/A	N/A	cultured human colorectal cancer cells (HCT116), and colorectal tissue samples	[122]
DNA framework supported electrochemical analysis	160 fg	*AR*, *CCNA1*, *CHFR*, *GSTP1*, *PTGS2*, *RASSF1*, *RPRM*, *SFA*, *SFRP1*, *TNFRSF10D*, *TGP1*, *and TIG1*	Prostate cancer cell line, cancer tissues, serum from cancer and BPH samples, and serum from normal samples	[123]
Assembly of a supersandwich electrochemical biosensor	450 aM	N/A	Hela cervical cancer cell line, PC-3 (epithelial cell line from a human prostatic adenocarcinoma), MCF-7 (breast cancer cell line)	[124]
AgNPs ^8^-decorated carbon nanotubes strategy	0.03 U/mL	N/A	Human serum	[125]
Methyl CpG-binding protein and glucose dehydrogenase-fused zinc finger protein	10^6^ copies	Androgen receptor promoter region	LNCaP, Du145 cells lines	[126]
Modified reduced graphene oxide platform	0.06 U/mL	N/A	Human serum	[127]

^1^ QD—quantum dot, FRET—Fluorescence Resonance Energy Transfer. ^2^ LCR—Ligation Chain Reaction, AuNP—Gold nanoparticle. ^3^ RE-EXPAR—Restriction Enzyme Exponential Amplification Reaction. ^4^ MELZA—Methylated DNA precipitation combined luciferase-fused zinc finger assay. ^5^ PG-EXPA—Primer generation exponential isothermal amplification-induced G-quadruplex formation. ^6^ In case of human blood (serum, plasma) samples with no target gene reference (N/A), the goal was to analyze methylation of circulating DNA. ^7^ ESCC—esophageal squamous cell carcinoma. ^8^ AgNP—silver nanoparticle.

### 4.2. Electrochemical Biosensors

EC biosensors monitor changes of selected electrical parameters, most often electric current (amperometric or voltammetric biosensors), potential (potentiometric biosensors), or resistance (impedimetric biosensors), before and after the biorecognition of the target analyte. They offer advantages of high sensitivity and specificity, low cost, simple operation and handling, miniaturization and rapid response times. EC biosensors comprise working and reference electrode (in two-electrode setup), or more often also a counter electrode in a three-electrode setup. The working electrode serves as a platform for the detection of the analyte and produces the response in the form of electrical signals. There is a plethora of EC approaches to study DNA methylation, reviewed, e.g., in [128,129,130]. Here, we describe only few recent examples; more works can be found in Table 1. For instance, Wang et al. developed an EC biosensor using target-induced conformation change of a DNA probe and exonuclease II-assisted target recycling [131]. In this approach, a stem-loop probe DNA was designed by conjugating a thiol group at the 3′-end that bound to Au nanoparticles-modified electrode via an Au-S bond, and methylene blue tag at 5′-end of the probe for signal readout. Then, the auxiliary DNA was introduced, opening the stem-loop probe DNA structure to form a dsDNA probe/auxiliary DNA, and moving the tag away from the electrode surface. After bisulfite treatment, 5mC in target DNA remained unchanged, easily hybridizing with auxiliary DNA which was later recycled and cleaved after the introduction of Exonuclease III (ExoIII). On the other hand, unmethylated C in target DNA was converted to U after bisulfite treatment, modifying the restriction site, and thus preventing its cleavage by ExoIII. This led to a weak current response for unmethylated target while methylated DNA produced higher current. Again, authors only simulated real sample environment by spiking 20% human serum with methylated target DNA, which is a common but insufficient feature of many biosensors.

An interesting EC biosensor based on paired-end tagging bisulfite amplification strategy was developed by Zhao’s group [119] (Figure 2B). Methylated, but not unmethylated DNA, was amplified by asymmetric MS-PCR using biotin-labeled reverse primer to generate abundant biotin-labeled ssDNA amplicons. DNA nanostructure in a form of self-assembled tetrahedral DNA immobilized at the gold electrode surface was used to capture these amplicons. Then, avidin-horseradish peroxidase (HRP) was introduced to bind to the biotin, producing the EC signal. This study included analysis of circulating DNA extracted from 200 microliters of plasma from eleven NSCLC patients. All eleven patient samples produced relatively higher current values than those of the healthy volunteer and the negative control, confirming the high methylation level of circulating DNA in these patients. However, using only one healthy control compared to eleven NSCLC patients is unusually low number.

Another approach, based on 5mC antibody, was utilized by Campuzano group. They reported an EC affinity-based biosensor for fast detection of gene specific methylations without a need of bisulfite treatment and PCR amplification [132] (Figure 2C.). In this work, two strategies were employed. First strategy in immunosensor format used two different antibodies; primary antibody against 5mC was immobilized onto the surface of carboxylic acid-modified magnetic beads, capable of capturing a methylated ssDNA. Secondary antibody conjugated with HRP (HRP-anti-ssDNA) targeted a captured ssDNA-antibody complex, and enzymatic reaction from HRP was monitored amperometrically on screen-printed electrodes. Second strategy was a DNA biosensor, using biotinylated capture probe immobilized onto surface of streptavidin-modified magnetic beads. This probe captured a target DNA; if the DNA was methylated, anti-5mC antibody recognized and bound to the 5mC, followed by an addition of a secondary antibody conjugated to HRP. Again, current from enzymatic reaction was monitored amperometrically. The DNA sensor exhibited higher sensitivity and allowed the detection of the gene-specific methylations; on the other hand, immunosensor better detected global DNA methylation. In addition, the DNA sensor demonstrated successful applicability for 1 h analysis of specific methylation in two relevant tumor suppressor genes in spiked biological fluids and in genomic DNA extracted from human glioblastoma cells. Later, the same group developed a similar biosensor for the detection of both 5mC and 5hmC at a global and gene-specific levels [120]. They applied the biosensor to analysis of global methylation level in paraffin-embedded colorectal tissues. Furthermore, they used it for locus specific methylation analysis of *MGMT* and *RASSF1A* tumor suppressor gene promoters in genomic DNA extracted from cancer cell lines, from paraffin-embedded colorectal tissues and also for cancer patient serum samples without previous DNA extraction.

These examples highlight a potential usefulness of DNA biosensors for methylation analysis, especially in terms of sensitivity, simplicity, low cost or rapid measurement times. However, most biosensors lack application to clinical samples and thus their feasibility is often difficult to evaluate. This is their major issue which needs to be overcome if biosensors want to serve as viable alternatives to standard methods of detection.

## 5. Conclusions

An importance of DNA methylation has started to emerge already in late 1970s, when a treatment of undifferentiated mouse embryo cell lines with a methylation inhibitor 5-azacytidine led to a formation of new differentiated cell phenotypes [133]. In 1980s, first papers appeared that showed altered methylation patterns in the DNA of cancer cells, i.e., both global hypomethylation along the genome [134] and localized hypermethylated CGIs within the gene promoter regions [135]. Soon after, researchers made a huge effort to elucidate mechanisms and functions of DNA methylation, starting with discovery of DNMT1 in 1988 as a DNA methylation writer [136], followed by discovery of DNMT3a and DNMT3b in 1998 [137], or recent finding of active erasers of DNA methylation, a TET family of proteins, in 2010 [138]. Interestingly, in early 1970s several groups showed that also RNA can be methylated (especially N6-methyl-adenosine), but the roles of this methylation are largely unknown and the identification of writers of this marker began very recently [139]. Since RNA methylation is out of scope of this review, readers can find more information in recent reviews [140,141]. Moreover, historic details can be found in recently published paper in IJMS describing timeline of epigenetic discoveries [142].

Along with these discoveries, more and more studies began to unveil a great importance of aberrant DNA methylation patterns across many types of solid tumors as well as in hematological malignancies [143]. Logically, this progress could not happen without a huge effort of researchers who modified existing analytical techniques or developed new ones to study DNA methylation. It soon became apparent that classical hybridization between two complementary DNA sequences will be insufficient, since both C and 5mC bind to G in exactly the same way. One solution to this problem lied in bisulfite-mediated specific deamination of cytosine discovered already in 1970 by two independent groups led by Shapiro [144] and Hayatsu [145]. These findings were later utilized by Frommer et al. who first devised a means to analyze 5mC in DNA [146]. Application of MSREs to analyze DNA methylation also dates back to late 1970s, when Cedar et al. directly detected 5mC in DNA using HpaII and MspI isoschizomers without requiring bisulfite treatment [76]. Both of these exciting discoveries as well as detailed timeline of various techniques that rely on them can be found in older review by Harrison and Parle-McDermott [147].

Despite an enormous progress in understanding the role of DNA methylation in cancer, only few methylated genes serve today as clinically relevant cancer biomarkers. These comprise, e.g., hypermethylated *SEPT9* in DNA extracted from plasma of patients with colorectal cancer [148], abnormal methylation levels of *GSTP1* (glutathione S-transferase pi 1) detectable in urine of prostate cancer patients [149], or *MGMT* promoter with methylation level that is inversely correlated with patient’s response to temozolomide treatment [150]. Perhaps the reasons for such a low number may lie in a fact that standard methods of detection are still time-consuming and relatively expensive, while biosensor-based alternatives are still nowhere close to being routinely used due to a lack of clinical data. Importantly, many studies still do not include large cohort of patients to successfully validate the biomarker or to show feasibility of newly developed technique, a step which is absolutely necessary but also slow and expensive. However, many companies now focus on developing DNA methylation-based IVD (in vitro diagnostics) assays and liquid biopsy tests using methods described in this review. A recent progress in translation of these IVD assays into clinic can be found in [29]. We may only hope that in near future, DNA methylation with its great potential to serve as a cancer biomarker, will fulfill these high expectations and will be routinely used for early diagnostics or to predict the outcome of cancer therapy.

## Abbreviations

5mC	5-methylcytosine
5hmC	5-hydroxymethylcytosine
AuNPs	Gold nanoparticle
bp	base pair
CGI	CpG island
Ct	Cycle threshold
COBRA	Combined bisulfite restriction analysis
DNMT	DNA methyltransferase
ELISA	Enzyme-Linked Immunosorbent Assay
EC	Electrochemical
HRCA	Hyperbranched rolling circle amplification
HPLC	High performance liquid chromatography
HPV	Human papillomavirus
HRP	Horseradish peroxidase
IVD	in vitro diagnostics
LUMA	Luminometric Methylation Assay
LOD	Limit of detection
MBD	Methyl-CpG-binding domain
MS-PCR	Methylation-specific PCR
MS-HRM	Methylation-specific high-resolution melting
MSRE	Methylation-sensitive restriction enzymes
MeDIP	Methylated DNA immunoprecipitation
MIRA	Methylated-CpG island recovery assay
MS-SnuPE	Methylation-sensitive single-nucleotide primer extension
MS-MLPA	Methylation-sensitive multiplex ligation-dependent probe amplification
MALDI-TOF	Matrix-assisted laser desorption/ionization time-of-flight
MS-MRM	Mass spectrometry multiple reaction monitoring
NGS	Next-generation sequencing
NSCLC	Non-small cell lung carcinoma
nt	nucleotide
OD	Optical density
RE	Restriction enzyme
RRBS	Reduced representation bisulfite sequencing
SNP	Single nucleotide polymorphism
SMRT	Single molecule real-time sequencing
WGBS	Whole genome bisulfite sequencing

## References

[1] Schübeler D. (2015). Function and information content of DNA methylation. Nature.

[2] Jin Z., Liu Y. (2018). DNA methylation in human diseases. Genes Dis..

[3] Schübeler D. (2015). ESCI award lecture: Regulation, function and biomarker potential of DNA methylation. Eur. J. Clin. Investig..

[4] Weber M., Hellmann I., Stadler M.B., Ramos L., Pääbo S., Rebhan M., Schübeler D. (2007). Distribution, silencing potential and evolutionary impact of promoter DNA methylation in the human genome. Nat. Genet..

[5] Nan X., Ng H.H., Johnson C.A., Laherty C.D., Turner B.M., Eisenman R.N., Bird A. (1998). Transcriptional repression by the methyl-CpG-binding protein MeCP2 involves a histone deacetylase complex. Nature.

[6] Liao J., Karnik R., Gu H., Ziller M.J., Clement K., Tsankov A.M., Akopian V., Gifford C.A., Donaghey J., Galonska C. (2015). Targeted disruption of DNMT1, DNMT3A and DNMT3B in human embryonic stem cells. Nat. Genet..

[7] Tahiliani M., Koh K.P., Shen Y., Pastor W.A., Bandukwala H., Brudno Y., Agarwal S., Iyer L.M., Liu D.R., Aravind L. (2009). Conversion of 5-Methylcytosine to 5-Hydroxymethylcytosine in Mammalian DNA by MLL Partner TET1. Science.

[8] You Jueng S., Jones Peter A. (2012). Cancer Genetics and Epigenetics: Two Sides of the Same Coin?. Cancer Cell.

[9] Kulis M., Esteller M., Herceg Z., Ushijima T. (2010). 2—DNA Methylation and Cancer. Adv Genet.

[10] Das P.M., Singal R. (2004). DNA methylation and cancer. J. Clin. Oncol..

[11] Li J., Poi M.J., Tsai M.-D. (2011). Regulatory Mechanisms of Tumor Suppressor P16INK4A and Their Relevance to Cancer. Biochemistry.

[12] Yu J., Ni M., Xu J., Zhang H., Gao B., Gu J., Chen J., Zhang L., Wu M., Zhen S. (2002). Methylation profiling of twenty promoter-CpG islands of genes which may contribute to hepatocellular carcinogenesis. BMC Cancer.

[13] Bearzatto A., Conte D., Frattini M., Zaffaroni N., Andriani F., Balestra D., Tavecchio L., Daidone M.G., Sozzi G. (2002). p16(INK4A) Hypermethylation detected by fluorescent methylation-specific PCR in plasmas from non-small cell lung cancer. Clin. Cancer Res..

[14] Esteller M., Corn P.G., Baylin S.B., Herman J.G. (2001). A gene hypermethylation profile of human cancer. Cancer Res..

[15] Sterlacci W., Tzankov A., Veits L., Zelger B., Bihl M.P., Foerster A., Augustin F., Fiegl M., Savic S. (2011). A Comprehensive Analysis of p16 Expression, Gene Status, and Promoter Hypermethylation In Surgically Resected Non-small Cell Lung Carcinomas. J. Thorac Oncol.

[16] Catteau A., Harris W.H., Xu C.-F., Solomon E. (1999). Methylation of the BRCA1 promoter region in sporadic breast and ovarian cancer: Correlation with disease characteristics. Oncogene.

[17] Chang P.-Y., Liao Y.-P., Wang H.-C., Chen Y.-C., Huang R.-L., Wang Y.-C., Yuan C.-C., Lai H.-C. (2017). An epigenetic signature of adhesion molecules predicts poor prognosis of ovarian cancer patients. Oncotarget.

[18] Lee M.G., Huh J.S., Chung S.K., Lee J.H., Byun D.S., Ryu B.K., Kang M.J., Chae K.S., Lee S.J., Lee C.H. (2006). Promoter CpG hypermethylation and downregulation of XAF1 expression in human urogenital malignancies: Implication for attenuated p53 response to apoptotic stresses. Oncogene.

[19] Martinez R., Setien F., Voelter C., Casado S., Quesada M.P., Schackert G., Esteller M. (2007). CpG island promoter hypermethylation of the pro-apoptotic gene caspase-8 is a common hallmark of relapsed glioblastoma multiforme. Carcinogenesis.

[20] Glaich O., Parikh S., Bell R.E., Mekahel K., Donyo M., Leader Y., Shayevitch R., Sheinboim D., Yannai S., Hollander D. (2019). DNA methylation directs microRNA biogenesis in mammalian cells. Nat. Commun.

[21] Hoffmann M.J., Schulz W.A. (2005). Causes and consequences of DNA hypomethylation in human cancer. Biochem. Cell Biol..

[22] Ross J.P., Rand K.N., Molloy P.L. (2010). Hypomethylation of repeated DNA sequences in cancer. Epigenomics.

[23] Tsuda H., Takarabe T., Kanai Y., Fukutomi T., Hirohashi S. (2002). Correlation of DNA hypomethylation at pericentromeric heterochromatin regions of chromosomes 16 and 1 with histological features and chromosomal abnormalities of human breast carcinomas. Am. J. Pathol.

[24] Colemon A., Harris T.M., Ramanathan S. (2020). DNA hypomethylation drives changes in MAGE-A gene expression resulting in alteration of proliferative status of cells. Gene Environ..

[25] Poojary M., Jishnu P.V., Kabekkodu S.P. (2020). Prognostic Value of Melanoma-Associated Antigen-A (MAGE-A) Gene Expression in Various Human Cancers: A Systematic Review and Meta-analysis of 7428 Patients and 44 Studies. Mol. Diagn Ther..

[26] Ekanayake Weeramange C., Tang K.D., Vasani S., Langton-Lockton J., Kenny L., Punyadeera C. (2020). DNA Methylation Changes in Human Papillomavirus-Driven Head and Neck Cancers. Cells.

[27] Hublarova P., Hrstka R., Rotterova P., Rotter L., Coupkova M., Badal V., Nenutil R., Vojtesek B. (2009). Prediction of Human Papillomavirus 16 E6 Gene Expression and Cervical Intraepithelial Neoplasia Progression by Methylation Status. Int. J. Gyn Cancer.

[28] Han H., Cortez C.C., Yang X., Nichols P.W., Jones P.A., Liang G. (2011). DNA methylation directly silences genes with non-CpG island promoters and establishes a nucleosome occupied promoter. Hum. Mol. Genet..

[29] Locke W.J., Guanzon D., Ma C., Liew Y.J., Duesing K.R., Fung K.Y.C., Ross J.P. (2019). DNA Methylation Cancer Biomarkers: Translation to the Clinic. Front. Genet..

[30] Taryma-Leśniak O., Sokolowska K.E., Wojdacz T.K. (2020). Current status of development of methylation biomarkers for in vitro diagnostic IVD applications. Clin. Epigenetics.

[31] Warnecke P.M., Stirzaker C., Song J., Grunau C., Melki J.R., Clark S.J. (2002). Identification and resolution of artifacts in bisulfite sequencing. Methods.

[32] Worm Ørntoft M.B., Jensen S., Hansen T.B., Bramsen J.B., Andersen C.L. (2017). Comparative analysis of 12 different kits for bisulfite conversion of circulating cell-free DNA. Epigenetics.

[33] Kint S., De Spiegelaere W., De Kesel J., Vandekerckhove L., Van Criekinge W. (2018). Evaluation of bisulfite kits for DNA methylation profiling in terms of DNA fragmentation and DNA recovery using digital PCR. PLoS ONE.

[34] Tierling S., Schmitt B., Walter J. (2018). Comprehensive Evaluation of Commercial Bisulfite-Based DNA Methylation Kits and Development of an Alternative Protocol With Improved Conversion Performance. Genet. Epigenet.

[35] Wang H., Ke H., Zheng Y., Lai J., Luo Q., Chen Q. (2017). A modified bisulfite conversion method for the detection of DNA methylation. Epigenomics.

[36] Qiu P., Soder G.J., Sanfiorenzo V.J., Wang L., Greene J.R., Fritz M.A., Cai X.Y. (2003). Quantification of single nucleotide polymorphisms by automated DNA sequencing. Biochem. Biophys. Res. Commun.

[37] Jiang M., Zhang Y., Fei J., Chang X., Fan W., Qian X., Zhang T., Lu D. (2010). Rapid quantification of DNA methylation by measuring relative peak heights in direct bisulfite-PCR sequencing traces. Lab. Invest..

[38] Brisotto G., di Gennaro A., Damiano V., Armellin M., Perin T., Maestro R., Santarosa M. (2015). An improved sequencing-based strategy to estimate locus-specific DNA methylation. BMC Cancer.

[39] Zhang Y., Rohde C., Tierling S., Stamerjohanns H., Reinhardt R., Walter J., Jeltsch A. (2009). DNA methylation analysis by bisulfite conversion, cloning, and sequencing of individual clones. Methods Mol. Biol..

[40] Huang Z., Bassil C.F., Murphy S.K. (2013). Bisulfite sequencing of cloned alleles. Methods Mol. Biol..

[41] Esteller M., Sparks A., Toyota M., Sanchez-Cespedes M., Capella G., Peinado M.A., Gonzalez S., Tarafa G., Sidransky D., Meltzer S.J. (2000). Analysis of adenomatous polyposis coli promoter hypermethylation in human cancer. Cancer Res..

[42] Stirzaker C., Millar D.S., Paul C.L., Warnecke P.M., Harrison J., Vincent P.C., Frommer M., Clark S.J. (1997). Extensive DNA methylation spanning the Rb promoter in retinoblastoma tumors. Cancer Res..

[43] Xu H., Wang W., Zhao J., Li T., Kang X. (2019). Aberrant hTERT promoter methylation predicts prognosis in Chinese patients with acral and mucosal melanoma: A CONSORT-compliant article. Medicine.

[44] Li Q., Gao H., Yang H., Wei W., Jiang Y. (2019). Estradiol promotes the progression of ER+ breast cancer through methylation-mediated RSK4 inactivation. Onco Targets Ther..

[45] Bassil C.F., Huang Z., Murphy S.K. (2013). Bisulfite pyrosequencing. Methods Mol. Biol..

[46] Harrington C.T., Lin E.I., Olson M.T., Eshleman J.R. (2013). Fundamentals of pyrosequencing. Arch. Pathol. Lab. Med..

[47] Dupont J.M., Tost J., Jammes H., Gut I.G. (2004). De novo quantitative bisulfite sequencing using the pyrosequencing technology. Anal. Biochem..

[48] Kreutz M., Hochstein N., Kaiser J., Narz F., Peist R. (2013). Pyrosequencing: Powerful and quantitative sequencing technology. Curr. Protoc. Mol. Biol..

[49] Delaney C., Garg S.K., Yung R. (2015). Analysis of DNA Methylation by Pyrosequencing. Methods Mol. Biol..

[50] Šestáková Š., Šálek C., Remešová H. (2019). DNA Methylation Validation Methods: A Coherent Review with Practical Comparison. Biol. Proced Online.

[51] https://www.epigendx.com/d/service/pyrosequencing.

[52] Ramalho-Carvalho J., Henrique R., Jerónimo C. (2018). Methylation-Specific PCR. Methods Mol. Biol..

[53] Eads C.A., Danenberg K.D., Kawakami K., Saltz L.B., Blake C., Shibata D., Danenberg P.V., Laird P.W. (2000). MethyLight: A high-throughput assay to measure DNA methylation. Nucleic Acids Res..

[54] Thomassin H., Kress C., Grange T. (2004). MethylQuant: A sensitive method for quantifying methylation of specific cytosines within the genome. Nucleic Acids Res..

[55] Dugast-Darzacq C., Grange T. (2009). MethylQuant: A real-time PCR-based method to quantify DNA methylation at single specific cytosines. Methods Mol. Biol..

[56] Wojdacz T.K., Dobrovic A., Hansen L.L. (2008). Methylation-sensitive high-resolution melting. Nat. Protoc..

[57] Hussmann D., Hansen L.L. (2018). Methylation-Sensitive High Resolution Melting (MS-HRM). Methods Mol. Biol..

[58] Majchrzak-Celińska A., Dybska E., Barciszewska A.M. (2020). DNA methylation analysis with methylation-sensitive high-resolution melting (MS-HRM) reveals gene panel for glioma characteristics. CNS Neurosci. Ther..

[59] Xiong Z., Laird P.W. (1997). COBRA: A sensitive and quantitative DNA methylation assay. Nucleic Acids Res..

[60] Bilichak A., Kovalchuk I. (2017). The Combined Bisulfite Restriction Analysis (COBRA) Assay for the Analysis of Locus-Specific Changes in Methylation Patterns. Methods Mol. Biol..

[61] Yang C.H., Chuang L.Y., Cheng Y.H., Gu D.L., Chen C.H., Chang H.W. (2010). Methyl-Typing: An improved and visualized COBRA software for epigenomic studies. FEBS Lett..

[62] Liu D., Enriquez L., Ford C.E. (2021). ROR2 Is Epigenetically Regulated in Endometrial Cancer. Cancers.

[63] Picketts D.J., Cameron C., Taylor S.A., Deugau K.V., Lillicrap D.P. (1992). Differential termination of primer extension: A novel, quantifiable method for detection of point mutations. Hum. Genet..

[64] Gonzalgo M.L., Jones P.A. (1997). Rapid quantitation of methylation differences at specific sites using methylation-sensitive single nucleotide primer extension (Ms-SNuPE). Nucleic Acids Res..

[65] Gonzalgo M.L., Jones P.A. (2002). Quantitative methylation analysis using methylation-sensitive single-nucleotide primer extension (Ms-SNuPE). Methods.

[66] Gonzalgo M.L., Liang G. (2007). Methylation-sensitive single-nucleotide primer extension (Ms-SNuPE) for quantitative measurement of DNA methylation. Nat. Protoc..

[67] El-Maarri O., Herbiniaux U., Walter J., Oldenburg J. (2002). A rapid, quantitative, non-radioactive bisulfite-SNuPE- IP RP HPLC assay for methylation analysis at specific CpG sites. Nucleic Acids Res..

[68] Xu X.H., Bao Y., Wang X., Yan F., Guo S., Ma Y., Xu D., Jin L., Xu J., Wang J. (2018). Hypoxic-stabilized EPAS1 proteins transactivate DNMT1 and cause promoter hypermethylation and transcription inhibition of EPAS1 in non-small cell lung cancer. FASEB J..

[69] Guo S., Yan F., Xu J., Bao Y., Zhu J., Wang X., Wu J., Li Y., Pu W., Liu Y. (2015). Identification and validation of the methylation biomarkers of non-small cell lung cancer (NSCLC). Clin. Epigenetics.

[70] Ehrich M., Nelson M.R., Stanssens P., Zabeau M., Liloglou T., Xinarianos G., Cantor C.R., Field J.K., van den Boom D. (2005). Quantitative high-throughput analysis of DNA methylation patterns by base-specific cleavage and mass spectrometry. Proc. Natl. Acad. Sci. USA.

[71] Coolen M.W., Statham A.L., Gardiner-Garden M., Clark S.J. (2007). Genomic profiling of CpG methylation and allelic specificity using quantitative high-throughput mass spectrometry: Critical evaluation and improvements. Nucleic Acids Res..

[72] Kunze S. (2018). Quantitative Region-Specific DNA Methylation Analysis by the EpiTYPER™ Technology. Methods Mol. Biol..

[73] Zeng H., Wang Y., Wang Y., Zhang Y. (2021). XXYLT1 methylation contributes to the occurrence of lung adenocarcinoma: Methylation and lung adenocarcinoma. Medicine.

[74] Siqueira J.F., Fouad A.F., Rôças I.N. (2012). Pyrosequencing as a tool for better understanding of human microbiomes. J. Oral Microbiol..

[75] Helmsauer K., Valieva M.E., Ali S., Chamorro González R., Schöpflin R., Röefzaad C., Bei Y., Dorado Garcia H., Rodriguez-Fos E., Puiggròs M. (2020). Enhancer hijacking determines extrachromosomal circular MYCN amplicon architecture in neuroblastoma. Nat. Commun..

[76] Cedar H., Solage A., Glaser G., Razin A. (1979). Direct detection of methylated cytosine in DNA by use of the restriction enzyme MspI. Nucleic Acids Res..

[77] Moore T. (2001). Southern analysis using methyl-sensitive restriction enzymes. Methods Mol. Biol..

[78] Hashimoto K., Kokubun S., Itoi E., Roach H.I. (2007). Improved quantification of DNA methylation using methylation-sensitive restriction enzymes and real-time PCR. Epigenetics.

[79] Nygren A.O., Ameziane N., Duarte H.M., Vijzelaar R.N., Waisfisz Q., Hess C.J., Schouten J.P., Errami A. (2005). Methylation-specific MLPA (MS-MLPA): Simultaneous detection of CpG methylation and copy number changes of up to 40 sequences. Nucleic Acids Res..

[80] Moelans C.B., Atanesyan L., Savola S.P., van Diest P.J. (2018). Methylation-Specific Multiplex Ligation-Dependent Probe Amplification (MS-MLPA). Methods Mol. Biol..

[81] Cross S.H., Charlton J.A., Nan X., Bird A.P. (1994). Purification of CpG islands using a methylated DNA binding column. Nat. Genet..

[82] Mohn F., Weber M., Schübeler D., Roloff T.C. (2009). Methylated DNA immunoprecipitation (MeDIP). Methods Mol. Biol..

[83] Kurdyukov S., Bullock M. (2016). DNA Methylation Analysis: Choosing the Right Method. Biology.

[84] Mitchell N., Deangelis J.T., Tollefsbol T.O. (2011). Methylated-CpG Island Recovery Assay. Methods Mol. Biol..

[85] Skvortsova K., Stirzaker C., Taberlay P. (2019). The DNA methylation landscape in cancer. Essays Biochem..

[86] Fernandez A.F., Valledor L., Vallejo F., Cañal M.J., Fraga M.F. (2018). Quantification of Global DNA Methylation Levels by Mass Spectrometry. Methods Mol. Biol..

[87] Berdasco M., Fraga M.F., Esteller M. (2009). Quantification of global DNA methylation by capillary electrophoresis and mass spectrometry. Methods Mol. Biol..

[88] Karimi M., Johansson S., Stach D., Corcoran M., Grandér D., Schalling M., Bakalkin G., Lyko F., Larsson C., Ekström T.J. (2006). LUMA (LUminometric Methylation Assay)—A high throughput method to the analysis of genomic DNA methylation. Exp. Cell Res..

[89] Karimi M., Johansson S., Ekström T.J. (2006). Using LUMA: A Luminometric-based assay for global DNA-methylation. Epigenetics.

[90] Ball M.P., Li J.B., Gao Y., Lee J.H., LeProust E.M., Park I.H., Xie B., Daley G.Q., Church G.M. (2009). Targeted and genome-scale strategies reveal gene-body methylation signatures in human cells. Nat. Biotechnol..

[91] Ito S., Shen L., Dai Q., Wu S.C., Collins L.B., Swenberg J.A., He C., Zhang Y. (2011). Tet proteins can convert 5-methylcytosine to 5-formylcytosine and 5-carboxylcytosine. Science.

[92] Zhang D., Wang Y., Bai Y., Ge Q., Qiao Y., Luo J., Jia C., Lu Z. (2008). A novel method to quantify local CpG methylation density by regional methylation elongation assay on microarray. BMC Genom..

[93] Smith Z.D., Gu H., Bock C., Gnirke A., Meissner A. (2009). High-throughput bisulfite sequencing in mammalian genomes. Methods.

[94] Taiwo O., Wilson G.A., Morris T., Seisenberger S., Reik W., Pearce D., Beck S., Butcher L.M. (2012). Methylome analysis using MeDIP-seq with low DNA concentrations. Nat. Protoc..

[95] Weber M., Davies J.J., Wittig D., Oakeley E.J., Haase M., Lam W.L., Schübeler D. (2005). Chromosome-wide and promoter-specific analyses identify sites of differential DNA methylation in normal and transformed human cells. Nat. Genet..

[96] Eid J., Fehr A., Gray J., Luong K., Lyle J., Otto G., Peluso P., Rank D., Baybayan P., Bettman B. (2009). Real-time DNA sequencing from single polymerase molecules. Science.

[97] Clark T.A., Murray I.A., Morgan R.D., Kislyuk A.O., Spittle K.E., Boitano M., Fomenkov A., Roberts R.J., Korlach J. (2012). Characterization of DNA methyltransferase specificities using single-molecule, real-time DNA sequencing. Nucleic Acids Res..

[98] Flusberg B.A., Webster D.R., Lee J.H., Travers K.J., Olivares E.C., Clark T.A., Korlach J., Turner S.W. (2010). Direct detection of DNA methylation during single-molecule, real-time sequencing. Nat. Methods.

[99] Beaulaurier J., Zhang X.S., Zhu S., Sebra R., Rosenbluh C., Deikus G., Shen N., Munera D., Waldor M.K., Chess A. (2015). Single molecule-level detection and long read-based phasing of epigenetic variations in bacterial methylomes. Nat. Commun..

[100] Laver T., Harrison J., O’Neill P.A., Moore K., Farbos A., Paszkiewicz K., Studholme D.J. (2015). Assessing the performance of the Oxford Nanopore Technologies MinION. Biomol. Detect. Quantif..

[101] Kono N., Arakawa K. (2019). Nanopore sequencing: Review of potential applications in functional genomics. Dev. Growth Differ..

[102] Petersen L.M., Martin I.W., Moschetti W.E., Kershaw C.M., Tsongalis G.J. (2019). Third-Generation Sequencing in the Clinical Laboratory: Exploring the Advantages and Challenges of Nanopore Sequencing. J. Clin. Microbiol..

[103] Rang F.J., Kloosterman W.P., de Ridder J. (2018). From squiggle to basepair: Computational approaches for improving nanopore sequencing read accuracy. Genome Biol..

[104] Ding H., Bailey A.D., Jain M., Olsen H., Paten B. (2020). Gaussian mixture model-based unsupervised nucleotide modification number detection using nanopore-sequencing readouts. Bioinformatics.

[105] Ni P., Huang N., Zhang Z., Wang D.P., Liang F., Miao Y., Xiao C.L., Luo F., Wang J. (2019). DeepSignal: Detecting DNA methylation state from Nanopore sequencing reads using deep-learning. Bioinformatics.

[106] Jayanthi V.S.P.K., Sankara A., Das A.B., Saxena U. (2017). Recent advances in biosensor development for the detection of cancer biomarkers. Biosens. Bioelectron..

[107] Khanmohammadi A., Aghaie A., Vahedi E., Qazvini A., Ghanei M., Afkhami A., Hajian A., Bagheri H. (2020). Electrochemical biosensors for the detection of lung cancer biomarkers: A review. Talanta.

[108] Li J., Li S., Yang C.F. (2012). Electrochemical Biosensors for Cancer Biomarker Detection. Electroanalysis.

[109] Li Z.-M., Pi T., Yan X.-L., Tang X.-M., Deng R.-H., Zheng X.-J. (2020). Label-free and enzyme-free one-step rapid colorimetric detection of DNA methylation based on unmodified gold nanoparticles. Spectrochim Acta A Mol. Biomol. Spectr..

[110] Cao A., Zhang C.-Y. (2012). Sensitive and Label-Free DNA Methylation Detection by Ligation-Mediated Hyperbranched Rolling Circle Amplification. Anal. Chem..

[111] Zhang Y., Hu J., Zou X., Ma F., Qiu J.-G., Zhang C.-Y. (2021). Integration of single-molecule detection with endonuclease IV-assisted signal amplification for sensitive DNA methylation assay. Chem. Commun..

[112] Ma Y., Zhang H., Liu F., Wu Z., Lu S., Jin Q., Zhao J., Zhong X., Mao H. (2015). Highly sensitive detection of DNA methylation levels by using a quantum dot-based FRET method. Nanoscale.

[113] Su F., Wang L., Sun Y., Liu C., Duan X., Li Z. (2015). Highly sensitive detection of CpG methylation in genomic DNA by AuNP-based colorimetric assay with ligase chain reaction. Chem. Commun..

[114] Wang Z.-Y., Wang L.-J., Zhang Q., Tang B., Zhang C.-Y. (2018). Single quantum dot-based nanosensor for sensitive detection of 5-methylcytosine at both CpG and non-CpG sites. Chem. Sci..

[115] Sun Y., Sun Y., Tian W., Liu C., Gao K., Li Z. (2018). A novel restriction endonuclease GlaI for rapid and highly sensitive detection of DNA methylation coupled with isothermal exponential amplification reaction. Chem. Sci..

[116] Hiraoka D., Yoshida W., Abe K., Wakeda H., Hata K., Ikebukuro K. (2012). Development of a Method To Measure DNA Methylation Levels by Using Methyl CpG-Binding Protein and Luciferase-Fused Zinc Finger Protein. Anal. Chem..

[117] Xue Q., Lv Y., Xu S., Zhang Y., Wang L., Li R., Yue Q., Li H., Gu X., Zhang S. (2015). Highly sensitive fluorescence assay of DNA methyltransferase activity by methylation-sensitive cleavage-based primer generation exponential isothermal amplification-induced G-quadruplex formation. Biosens. Bioelectron..

[118] Dadmehr M., Hosseini M., Hosseinkhani S., Reza Ganjali M., Sheikhnejad R. (2015). Label free colorimetric and fluorimetric direct detection of methylated DNA based on silver nanoclusters for cancer early diagnosis. Biosens. Bioelectron..

[119] Wang X., Chen F., Zhang D., Zhao Y., Wei J., Wang L., Song S., Fan C., Zhao Y. (2017). Single copy-sensitive electrochemical assay for circulating methylated DNA in clinical samples with ultrahigh specificity based on a sequential discrimination-amplification strategy. Chem. Sci..

[120] Povedano E., Montiel V.R.-V., Valverde A., Navarro-Villoslada F., Yáñez-Sedeño P., Pedrero M., Montero-Calle A., Barderas R., Peláez-García A., Mendiola M. (2019). Versatile Electroanalytical Bioplatforms for Simultaneous Determination of Cancer-Related DNA 5-Methyl- and 5-Hydroxymethyl-Cytosines at Global and Gene-Specific Levels in Human Serum and Tissues. ACS Sens..

[121] Haque M.H., Gopalan V., Yadav S., Islam M.N., Eftekhari E., Li Q., Carrascosa L.G., Nguyen N.-T., Lam A.K., Shiddiky M.J.A. (2017). Detection of regional DNA methylation using DNA-graphene affinity interactions. Biosens. Bioelectron..

[122] Furst A.L., Muren N.B., Hill M.G., Barton J.K. (2014). Label-free electrochemical detection of human methyltransferase from tumors. Proc. Natl. Acad. Sci. USA.

[123] Chen S., Su J., Zhao Z., Shao Y., Dou Y., Li F., Deng W., Shi J., Li Q., Zuo X. (2020). DNA Framework-Supported Electrochemical Analysis of DNA Methylation for Prostate Cancers. Nano Lett..

[124] Feng Q., Wang M., Qin L., Wang P. (2019). Dual-Signal Readout of DNA Methylation Status Based on the Assembly of a Supersandwich Electrochemical Biosensor without Enzymatic Reaction. ACS Sensors.

[125] Gao F., Fan T., Ou S., Wu J., Zhang X., Luo J., Li N., Yao Y., Mou Y., Liao X. (2018). Highly efficient electrochemical sensing platform for sensitive detection DNA methylation, and methyltransferase activity based on Ag NPs decorated carbon nanocubes. Biosens. Bioelectron..

[126] Lee J., Yoshida W., Abe K., Nakabayashi K., Wakeda H., Hata K., Marquette C.A., Blum L.J., Sode K., Ikebukuro K. (2017). Development of an electrochemical detection system for measuring DNA methylation levels using methyl CpG-binding protein and glucose dehydrogenase-fused zinc finger protein. Biosens. Bioelectron..

[127] Sedlackova E., Bytesnikova Z., Birgusova E., Svec P., Ashrafi A.M., Estrela P., Richtera L. (2020). Label-Free DNA Biosensor Using Modified Reduced Graphene Oxide Platform as a DNA Methylation Assay. Materials.

[128] Campuzano S., Pingarrón J.M. (2018). Electrochemical Sensing of Cancer-related Global and Locus-specific DNA Methylation Events. Electroanalysis.

[129] Zhang Q., Wu Y., Xu Q., Ma F., Zhang C.Y. (2021). Recent advances in biosensors for in vitro detection and in vivo imaging of DNA methylation. Biosens. Bioelectron..

[130] Bartosik M., Hrstka R. (2017). Bioelectrochemistry of nucleic acids for early cancer diagnostics—Analysis of DNA methylation and detection of microRNAs. Rev. Anal. Chem..

[131] Feng Q., Qin L., Wang M., Wang P. (2020). Signal-on electrochemical detection of DNA methylation based on the target-induced conformational change of a DNA probe and exonuclease III-assisted target recycling. Biosens. Bioelectron..

[132] Povedano E., Vargas E., Montiel V.R.-V., Torrente-Rodríguez R.M., Pedrero M., Barderas R., Segundo-Acosta P.S., Peláez-García A., Mendiola M., Hardisson D. (2018). Electrochemical affinity biosensors for fast detection of gene-specific methylations with no need for bisulfite and amplification treatments. Sci. Rep..

[133] Taylor S.M., Jones P.A. (1979). Multiple new phenotypes induced in 10T1/2 and 3T3 cells treated with 5-azacytidine. Cell.

[134] Feinberg A.P., Vogelstein B. (1983). Hypomethylation distinguishes genes of some human cancers from their normal counterparts. Nature.

[135] Baylin S.B., Höppener J.W., de Bustros A., Steenbergh P.H., Lips C.J., Nelkin B.D. (1986). DNA methylation patterns of the calcitonin gene in human lung cancers and lymphomas. Cancer Res..

[136] Bestor T., Laudano A., Mattaliano R., Ingram V. (1988). Cloning and sequencing of a cDNA encoding DNA methyltransferase of mouse cells. The carboxyl-terminal domain of the mammalian enzymes is related to bacterial restriction methyltransferases. J. Mol. Biol..

[137] Okano M., Bell D.W., Haber D.A., Li E. (1999). DNA Methyltransferases Dnmt3a and Dnmt3b Are Essential for De Novo Methylation and Mammalian Development. Cell.

[138] Ito S., D’Alessio A.C., Taranova O.V., Hong K., Sowers L.C., Zhang Y. (2010). Role of Tet proteins in 5mC to 5hmC conversion, ES-cell self-renewal and inner cell mass specification. Nature.

[139] Dubin D.T., Taylor R.H. (1975). The methylation state of poly A-containing-messenger RNA from cultured hamster cells. Nucleic Acids Res..

[140] Zaccara S., Ries R.J., Jaffrey S.R. (2019). Reading, writing and erasing mRNA methylation. Nat. Rev. Mol. Cell Biol..

[141] Zhou Y., Kong Y., Fan W., Tao T., Xiao Q., Li N., Zhu X. (2020). Principles of RNA methylation and their implications for biology and medicine. Biomed. Pharmacother..

[142] Peixoto P., Cartron P.-F., Serandour A.A., Hervouet E. (2020). From 1957 to Nowadays: A Brief History of Epigenetics. Int. J. Mol. Sci..

[143] Wajed S.A., Laird P.W., DeMeester T.R. (2001). DNA methylation: An alternative pathway to cancer. Ann. Surg..

[144] Shapiro R., Servis R.E., Welcher M. (1970). Reactions of Uracil and Cytosine Derivatives with Sodium Bisulfite. J. Am. Chem. Soc..

[145] Hayatsu H., Wataya Y., Kai K. (1970). Addition of sodium bisulfite to uracil and to cytosine. J. Am. Chem. Soc..

[146] Frommer M., McDonald L.E., Millar D.S., Collis C.M., Watt F., Grigg G.W., Molloy P.L., Paul C.L. (1992). A genomic sequencing protocol that yields a positive display of 5-methylcytosine residues in individual DNA strands. Proc. Natl. Acad. Sci. USA.

[147] Parle-Mcdermott A., Harrison A. (2011). DNA Methylation: A Timeline of Methods and Applications. Front. Genet..

[148] Grützmann R., Molnar B., Pilarsky C., Habermann J.K., Schlag P.M., Saeger H.D., Miehlke S., Stolz T., Model F., Roblick U.J. (2008). Sensitive detection of colorectal cancer in peripheral blood by septin 9 DNA methylation assay. PLoS ONE.

[149] Gonzalgo M.L., Pavlovich C.P., Lee S.M., Nelson W.G. (2003). Prostate cancer detection by GSTP1 methylation analysis of postbiopsy urine specimens. Clin. Cancer Res..

[150] Esteller M., Garcia-Foncillas J., Andion E., Goodman S.N., Hidalgo O.F., Vanaclocha V., Baylin S.B., Herman J.G. (2000). Inactivation of the DNA-repair gene MGMT and the clinical response of gliomas to alkylating agents. N. Engl. J. Med..

